# A Medication Safety Model: A Case Study in Thai Hospital

**DOI:** 10.5539/gjhs.v5n5p89

**Published:** 2013-06-12

**Authors:** Phichai Rattanarojsakul, Natcha Thawesaengskulthai

**Affiliations:** 1 Technopreneurship and Innovation Management Program, Chulalongkorn University, Bangkok, Thailand; 2 Department of Industrial Engineering, Faculty of Engineering, Chulalongkorn University, Bangkok, Thailand

**Keywords:** medication errors, drug errors, medication management, patient safety

## Abstract

Reaching zero defects is vital in medication service. Medication error can be reduced if the causes are recognized. The purpose of this study is to search for a conceptual framework of the causes of medication error in Thailand and to examine relationship between these factors and its importance. The study was carried out upon an in-depth case study and survey of hospital personals who were involved in the drug use process. The structured survey was based on Emergency Care Research Institute (ECRI) (2008) questionnaires focusing on the important factors that affect the medication safety. Additional questionnaires included content to the context of Thailand's private hospital, validated by five-hospital qualified experts. By correlation Pearson analysis, the result revealed 14 important factors showing a linear relationship with drug administration error except the medication reconciliation. By independent sample t-test, the administration error in the hospital was significantly related to external impact. The multiple regression analysis of the detail of medication administration also indicated the patient identification before administration of medication, detection of the risk of medication adverse effects and assurance of medication administration at the right time, dosage and route were statistically significant at 0.05 level. The major implication of the study is to propose a medication safety model in a Thai private hospital.

## 1. Introduction

During 1995 to 2010 The Joint Commission of the United States (2011) showed 6,782 serious adverse events and 67 percent resulted in death. Medication error was in the top ten of serious adverse events in 2010. Medication errors harm an estimated 1.5 million people in the United States each year and result in increased medical costs to treat adverse events from using the drug estimated at 5.3 million dollars per year (The Institute of Medicine of the United States, 2006). A survey of medication errors in 1,116 hospitals in the United States, the figure was 5.07 percent (Bond et al., 2001). In Europe, it was found that adverse events occurred at a cost of billions of pounds a year and 48-49 percent was caused by drugs and could be prevented (Taxis & Barber, UK and Germany, 2004). In Australia, adverse event occurred at a cost about 350 million U.S. dollars per year and 43 percent was caused by drugs and could be prevented (Hodgkinson, 2006). In Japan, it was found that 46.6 percent of the adverse event was caused by drugs and could also be prevented (Nakajima, 2005). In Thailand, there is no national data collection and this problem has never been studied in the economic sense.

Medication errors are caused by many factors during the medication administration. Reduction of human error was emphasized to be the major source to minimize errors in many literatures. Human error was associated with failure of action causing the deviation of doing what is right (Hansen, 2006). Kleinpell (2001) pointed out that many factors which lead to errors in medication use were associated with the human experience such as new or temporary staffs who were lack of knowledge and would trend to produce incorrect documentation. Noise and other factors such as fatigue, stress and indolence cause harm patients. These are factors associated with human error (Rassin et al., 2005). Although human error is the cause of medication error; it is believed to have little effect (Henneman & Gawlinski, 2004). Since human error is relatively easy to be recognized, it raises the culture of blaming human error in health care organizations (Institute of Medicine, 2001). Blame culture is an adverse effect on workers and certainly affects patients as well. Once confidence has been destroyed it will affect changes in patient care and the ability to work. The failure to find the exact cause of the error from the operating conditions may eventually causes more and repeated errors.

**Figure 1:**
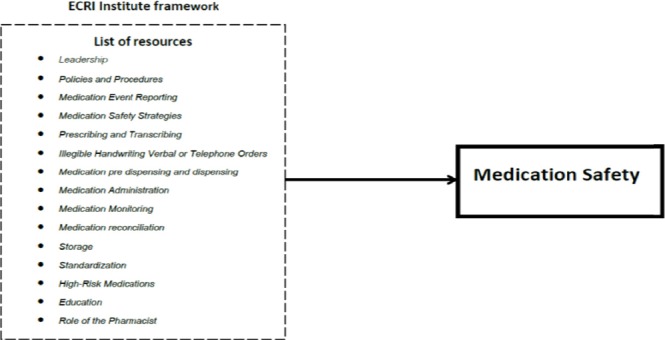
Medication safety (ECRI)

Brady and Fleming (2009) concluded five contributing factors to medication errors as barriers to report, knowledge & skill, deviation from procedures, reconciling medical history, prescriptions and drug distribution systems. The study of Jordan by Mrayyan et al. (2007) found that the causes of the medication error was related to defective drug labeling or packaging, the confusion of different types of injection device and noise at work. Teinila et al. (2011) found that the medication error was associated with five origins such as physician, organization, information technology, patient and hospital. Emergency Care Research Institute (ECRI Institute) (2008) has issued a set of self-assessment questionnaires for the hospital managers to assess the safety of drugs in the hospital (medication safety). It summarizes 15 important matters collected from leading medical institutions in the United States covering national and international medical errors (Figure 1). The definition of these factors (list of resource) is shown in Table 1.

**Table 1:** Definition of the factors (list of resource)

The factors	Definition
Leadership	Need to adequately address the demands of an ever changing health care environment for medication error reduction initiatives.
Policies and procedures	Medication Policy and related procedures have been developed to ensure the appropriate administration of medication
Storage of the drug	Many errors are preventable simply by minimizing floor stock, restricting access to high-alert drugs and hazardous chemicals, use commercially available solutions and standard concentrations to minimize error-prone processes.
Prescribing and transcribing	The prescription involves an action of a legitimate prescriber to issue a medication order. The transcription involves anything related to the act of transcribing an order (by nurse, pharmacist, or clerk) for order processing (e.g., electronically or manually in the patient's record).
Illegible handwriting, verbal or telephone orders	It is important to be alert for illegibility and to the prescription orally or by phone. Any doubt or confusion must be resolved before dispensing or administering the medicine.
Predispensing and dispensing medication	Predispensing activities include printed data, patient's name, drug name, drug use in the drug label. Dispensing activities include order review, entry/processing, preparation, and dispensation (including stocking automated dispensing devices).
Medication administration	Administering activities begin in the patient care unit, care delivery area, or patient bedside and continue through actual drug administration to the patient. It includes giving the right medication to the right patient at the right time and informing the patient about the medication.
Surveillance of drug monitoring	Monitoring activities involves evaluation of patient's physical, emotional, or psychological responses to the medication with record of such findings.
High-risk medications	High-alert medications are drugs that have a higher risk of causing significant patient harm when they are used erroneously.
Security strategy of the drug (Medication Safety Strategies)	Activities to deliver safe, effective and cost efficient use of medications
Medication reconciliation	Process in which healthcare providers work together with patients and the families to ensure accurate and comprehensive medication information and communicate consistently across transition of care. Medication reconciliation requires a systematic and comprehensive review of all the medications the patient is taking
Standardization	Extensive staff involvement and multiple iterations resulted in agreement on a single administration for each drug.
The role of pharmacist	Pharmacists are often assumed to be the “guardians” in ensuring that medication errors do not occur. This expectation is unrealistic, because avoiding error is a health care team effort.
Medication education	Education concerning new medications, nonformulary or high-alert medications, and medication error prevention
Medication event reporting	Staffs should be convinced in the local incident reporting system and to notify healthcare managers of medication incidents that are occurring, including near misses

Source: adapt from continuing care risk management, self-assessment questionnaires, ECRI Institute (2008)

In the context of Thai literature, Tewthanom and Thananonniwas (2007) proposed the concept that medication errors were caused either by individual or system. In individual concept, the persons will be accused and blamed from people around once the error occurs or they are obliged to undergo new training. Thus it is likely that the fault will be concealed or masked. This obliterates opportunity of disclosing the underlying error to avoid forthcoming mistake. In system concepts, the aim is to correct the surrounding environment not the human behavior.

Interview was conducted in five Thai healthcare qualified experts. The opinions were that the reduction of medication errors were also influenced by external impact such as the certification standard of the hospital and professional standard of the hospital staffs. The errors can be reduced when these factors are controlled.

Following the frame of self-assessment questionnaire of ECRI Institute (2008), causative factors of medication error with related authors were summarized in Table 2 and were applied in the proposed case study in this paper.

**Table 2:** Factors of medication errors: Authors

Factors contributing to medication errors	Authors
Leadership	Institute of Medicine [IOM] (2001), Khatri et al. (2006), Henneman & Gawlinsk (2004), Reason (1997), Hansen (2006), Kleinpell (2001), Tewthanom & Thananonniwas (2007)
Policies and Procedures	Hughes & Ortiz (2005); McIntyre & Courey (2007), Benner et al. (2002), Brady & Fleming (2009), Greenall & Wichman (2006), Teinila et al. (2011), Tewthanom & Thananonniwas (2007)
Storage	Greenall and Wichman (2006), Mrayyan et al. (2007), Teinila et al. (2011), Tewthanom & Thananonniwas (2007)
Prescribing and Transcribing	Fry and Darcy (2007), Teinila et al. (2011), Runciman et al. (2003), Hicks et al. (2004), Yupa et al. (2008), Tewthanom & Thananonniwas (2007)
Illegible Handwriting, Abbreviations, Verbal or Telephone Orders	Mayo & Duncan (2004), Fry & Darcy (2007), William (2004), Runciman et al. (2003)
Medication predispensing and dispensing	Madegowda et al. (2007), Hronek & Bleich (2002), Mrayyan et al. (2007), Yupa et al. (2008), Tewthanom & Thananonniwas (2007)
Medication Administration	Valentin et al. (2009), Teinila et al. (2011), Hicks et al. (2004), Tewthanom & Thananonniwas (2007)
Medication Monitoring	Teinila et al. (2011), Hicks et al. (2007), Tewthanom & Thananonniwas (2007)
High-Risk Medications	The Joint Commission (2006), Tewthanom & Thananonniwas (2007)
Medication Safety Strategies	Brady & Fleming (2009), Hronek & Bleich (2002), Bennett et al. (2006), Tewthanom and Thananonniwas (2007)
Medication reconciliation	Brady & Fleming (2009), Sullivan et al. (2005), The Joint Commission (2006)
Standardization	The Joint Commission (2006), Tewthanom & Thananonniwas (2007)
Role of the Pharmacist	Brady and Fleming (2009), Madegowda et al. (2007)
Education	Brady and Fleming (2009), UKCC (2000)
Medication Event Reporting	Brady and Fleming (2009)

Medication error can be considered as a mischief. Not only physically sick patients are affected, it also induces psychological effects that could not be virtually assessed in either patients or relatives. Medication error can be fatal, it can be a big suffering to the patients and a great loss of expenses which health care providers may have to face with litigation. Therefore, prevention of medication errors or to minimize the problem is extremely important.

In accordance to Stuart criteria, a private hospital was selected for this research. It is a well-known hospital with good performance records which provide representative information and thus is suitable for an investigation (Stuart et al., 2002). It collaborates to research and accessibility. The hospital is in the second largest hospital chains in Thailand with approximate annual income of 32 million USD (1,000 million Thai baht). It is a four- hundred beds hospital, where 39 percent of the adverse event were caused by preventable medication errors. Moreover, most medication errors are administration error. It therefor is justified to find out if there is any significant correlation between the factor variables and the medication errors. The result of this study will subsequently lead to issue a guideline for the hospital management team to cope with the medication error.

## 2. Purpose of the Study

**Figure 2:**
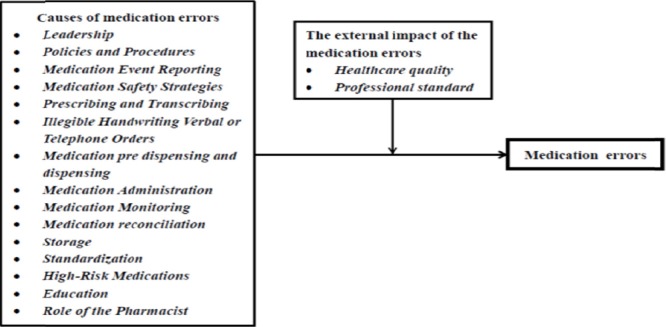
Preliminary framework of medication errors for Thai hospitals

To recognize the relationship of the important causative factors and the medication errors, a theoretical framework was developed and presented in Figure 2. The resultant information will be useful for Thai hospital management staffs who conduct medication safety policy. This information may be used for medication safety instruction by staffs and managers in the hospital, hospital accreditation institutes in Thailand and other organizations throughout the world.

Based on the stated purposes, the following research questions were formulated:

1). What are the levels of importance of these factors that affect the medication errors in a Thai private hospital?
2). How often do these factors implement were practiced to reduce medication errors?
3). How do these factors affect the most common type of medication errors?
4). What are the detail description of these factors that affect the most common type of medication errors?

## 3. Method

### 3.1 Sample and Data Collection

#### 3.1.1 Questionnaire

Questionnaire covering the causes and preliminary findings of medical error were presented to forty six hospital staffs during October to December 2012 (crossectional approach) for answering. The medication error is the dependent variable, and the set of the important factor variable is the independent variable. The direction of expected effects of these variables is indicated by arrows in Figure 2. It is proposed that these important factor variables would positively contribute to the medication errors. The questionnaire was divided into three sections: A) General information of the organization B) The prevention of medication errors and C) suggestions and comments. The respondents were required to complete all three sections. There were 8 questions in section A, 216 questions in section B and 2 questions in section C. Data were scored through questionnaire on 5 point Likert scale, 1=strongly disagree to 5=strongly agree. All the answers were received from 46 respondents.

There were two main objectives in designing the questionnaire. First was to maximise the proportion of population answering the questionnaire—that is, the response rate and to obtain accurate relevant information for the survey. Second was to maximise response rate-that is the author had to carefully consider the way questionnaires are administered, explaining the purpose of the survey, and keep reminding those who had not responded. In order to obtain accurate and relevant answer, the author was present at the interview session to explain and clarify any question which might arise.

#### 3.1.2 Case study Sampling

Case study sampling of the population was by randomization, regardless of the probability (non-probability) and sampling method specific (purposive sampling) due to sampling convenience and limitation. Critical case sampling is a type of purposive sampling technique that is particularly useful in exploratory qualitative research, which is of limited resources, as well as research where a single case (or small number of cases) can be concluded in explaining the phenomenon of interest. The case study selected a large hospital occupying more than 100 beds with a standard quality of hospital accreditation in Thailand (HA). Sources of primary data were obtained from 46 staffs who work with drug use process in the hospital, consisted of 26 general staffs in nursing department, 11 head-nurses in different sections, 2 managers of nursing department, 1 physician, 4 pharmacists, 1 hospital quality staff and 1 medical director. All the proposed factors affecting medication error variables were based on ECRI (2008) questionnaire and two additional factors from Thai experts’ opinion. The data for these variables were computed and used in data analysis.

### 3.2 Expert Interview and Validity

Five Thai healthcare quality experts were interviewed. Semi- structured interview was adapted for each expert. There were two quality directors from two private hospitals, one quality manager from a private hospital and another one from a public hospital, the last one was an expert surveyor from the Institute and Hospital Accreditation (HA) They were interviewed in order to get an actual and in-depth view of medication errors in Thai hospitals; In addition, 226 questions were constructed and presented to these five experts to verify the legitimacy by applying. [Item-Objective Congruency index (IOC)] Each question was rated in three scales: ‘-1’ representing disagreement, ‘0’ representing uncertainty, and ‘+1’ representing agreement. IOC index from all questions was 0.852 (n = 5) which illustrated the acceptable level of content validity (IOC index must more than 0.5)

### 3.3 The Reliability

Calculating Cronbach's alpha is the most commonly used method to estimate reliability. The questionnaires were constructed for this study to pilot test with target population of 46 persons who were involved in the hospital drug use process. The Cronbach's coefficient alpha of the significance of important factors affecting the cause of medication errors was 0.9942 and the actual implementation was 0.9964. Both demonstrated acceptable level of internal consistency.

### 3.4 Measurement of Variables

The organizational variables were measured as follows:

1). Dependent variable represents the medication errors.
2). Independent variables represent the proposed causes of medication error, adapted from ECRI (2008) framework and Thai expert opinion.

### 3.5 Statistical Analysis

The SPSS for Windows was used to analyze descriptive statistics, frequency distribution, percentage and standard deviation. The correlation coefficient of Pearson Product Moment coefficient correlation, independent samples t-test and stepwise multiple regression analysis were used to examine the research questions. Enter Regression analysis was used for variable selection technique and also the assumption of regression equations to determine all the possible relationships between independent and dependent variables.

## 4. Results

The results of the data analysis showed ten respondents from the ward (21.7%), and eight from the ICU (17.4%) consisting the two major department groups involved in the drug use process, though twelve respondents were from OPD department (26%). When the respondents were classified individually, they were twenty six nursing staffs (56.5%), eleven head nurses (23.9%) four pharmacists (8.7%), two managers of nursing department (4.3%), one physician (2.2%) as well as one medical director (2.2%).

Forty four respondents (95.7%) recognized the causes of medication error except two who did not (4.3%) Only forty two persons (91.3%) of these population recognized that they were preventable errors. Despite the fact that twenty eight personals (60.9%) expressed the lack of solution for prevention, the remaining eighteen respondents (39.1%) who admitted the existence of preventive measures but expressed that these measures were solely by avoid repeating the mistakes without a definitive solution. According to the author's view point, this is not an appropriate approach for prevention of errors.

### 4.1 The Common Types of Medication Error Are Shown in Table 3

**Table 3:** Type of medication error

Types of medication errors	N	Mean	Std. Deviation	Opinion	Rank
prescribing error	46	1.61	0.774	very few problems	4
transcribing error	46	1.83	0.797	a few problems	3
pre-dispensing error	46	1.52	0.722	very few problems	5
dispensing error	46	2.24	0.673	a few problems	2
administration error	46	4.52	0.505	many problems	1

**Figure 3:**
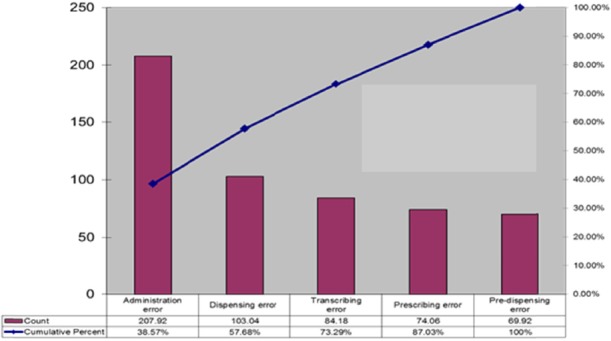
Problems in the drug use system

The highest mean was the administration error, reflecting the most severe problem in the drug use system. There were a few other problems including errors in copying drug orders (transcribing error) and in dispensation (dispensing error). The rarer errors were prescribing and pre-dispensing errors. Types of medication errors are shown in Pareto charts (Figure 3).

From Pareto chart, the three key medication error types are administration, dispensing and transcribing errors respectively. Thus, the administration error should be the priority in the process of renovation.

### 4.2 The External Impact of the Medication Errors

In this study, the external impacts were the certification standard and the professional standard of hospital staffs. The study revealed forty two respondents (93.3%) recognizing the prevention of medication errors affect hospital accreditation but four (8.7%) did not, whereas forty one persons recognized its influence to professional standard but five (10.9%) did not.

### 4.3 Results of the Research Questions

#### 4.3.1 Questions 1: What are the levels of importance of these factors affecting the medication errors in a Thai private hospital?

The study showed that all respondents expressed “strong agreement” with all the important factors and were ranked in descending order of importance as follows: prescribing and transcribing, illegible handwriting, abbreviations, verbal or telephone orders, high-risk medications, policies and procedures, medication monitoring, storage, medication administration, standardization, education, medication safety strategies, medication event reporting, medication predispensing and dispensing, role of the pharmacist, leadership and medication reconciliation respectively.

The significance of all the factors affecting medication error that were recognized by all respondents as “Strongly agreed”, suggesting these factors are to be strongly considered in the prevention of medication errors for hospitals in Thailand.

#### 4.3.2 Questions 2: How often do these factors implement were practiced to reduce medication errors?

The study showed that all respondents expressed “moderate practice” of all the important factors and the actual frequency of practice were ranked in descending order as follows: high-risk medications, standardization, medication administration, illegible handwriting, abbreviations, verbal or telephone orders, medication monitoring, storage, medication event reporting, prescribing and transcribing, medication safety strategies, medication predispensing and dispensing, education, policies and procedures, medication reconciliation, role of the pharmacist and leadership respectively. This reflected that these factors were not performed appropriately and the hospital should demand personals to practice strictly in order to reduce the administration error. By applying Paired-Samples Test, the results of research question 1 and question 2 are demonstrated in Table 4.

**Table 4:** Mean and Paired Samples test of medication error factors

The important factors	N	Mean		Paired Samples Test

		Important	Actual performance	Sig.(2-tailed)
Leadership	46	4.3512	2.6756	<0.001
Policies and Procedures	46	4.5185	2.7717	<0.001
Storage	46	4.4913	2.9159	<0.001
Prescribing and Transcribing	46	4.6232	2.8261	<0.001
Illegible Handwriting, Abbreviations Verbal or Telephone Orders	46	4.6155	2.928	<0.001
Medication pre dispensing and dispensing	46	4.3956	2.8181	<0.001
Medication Administration	46	4.4844	2.9545	<0.001
Medication Monitoring	46	4.5145	2.9203	<0.001
High-Risk Medications	46	4.5621	3.0714	<0.001
Medication Safety Strategies	46	4.4091	2.8194	<0.001
Medication reconciliation	46	4.3478	2.7174	<0.001
Standardization	46	4.4855	2.9674	<0.001
Role of the Pharmacist	46	4.3873	2.7174	<0.001
Education	46	4.4742	2.788	<0.001
Medication Event Reporting	46	4.403	2.8428	<0.001

Null hypothesis: There is no significant difference between the means of the two variables.

Alternate hypothesis: There is a significant difference between the means of the two variables

The last column showed the significant value of less than 0.05 which rejected the Null hypothesis. There is statistically significant difference at 95% confidence level (P <0.05), demonstrating a significant difference between the means of important and actual performances. This reflected that these factors were essential and should effectively implemented. In this hospital, these factors affecting medical errors were not cautiously practiced. Therefore, it is important to improve the performance of these hospital personals effectively to reduce the medication errors.

#### 4.3.3 Questions 3: How do these factors affect the most common type of medication errors?

The study revealed most respondents recognized that administration error carried multiple problems in the drug use system. Thus the most common type of medication errors in this hospital was the administration error.

It is believed that the independent variables (the 15 important factors by the theory of ECRI Institute (2008) affect medication errors. The concept of ECRI Institute (2008) was analysed concerning the correlation between independent variables and the dependent variable (administration error), to find out whether the independent variables was a linear relationship with the dependent variable, by applying Pearson correlation (Pearson Product Moment Correlation Coefficient) and the result was shown in Table 5.

**Table 5:** Correlation of independent and dependent variables

	Administration errors		
	Pearson Correlation	Sig. (2-tailed)	N
Leadership	0.571(**)	<0.001	46
Policies and Procedures	0.511(**)	<0.001	46
Storage	0.545(**)	<0.001	46
Prescribing and Transcribing	0.440(**)	0.002	46
Illegible Handwriting, Abbreviations Verbal or Telephone Orders	0.512(**)	<0.001	46
Medication pre dispensing and dispensing	0.419(**)	0.004	46
Medication Administration	0.467(**)	0.001	46
Medication Monitoring	0.452(**)	0.002	46
High-Risk Medications	0.372(*)	0.011	46
Medication Safety Strategies	0.364(*)	0.013	46
Medication reconciliation	0.267	0.073	46
Standardization	0.335(*)	0.023	46
Role of the Pharmacist	0.438(**)	0.002	46
Education	0.317(*)	0.032	46
Administration errors	1	.	46

* Correlation is significant at the 0.05 level (2-tailed).

** Correlation is significant at the 0.01 level (2-tailed).

Table 5 showed the relationship of these factors (Independent variables) to administration error (dependent variables) at the level of significance. Those factors with the level of statistical significance at 0.01 (2-tailed) and Pearson correlation of moderate to high percentage were the Leadership 57%, Policies and Procedures 51%, Storage 55%, Prescribing and Transcribing 44%, Illegible Handwriting, Abbreviations Verbal or Telephone Orders 51%, Medication predispensing and dispensing 42%, Medication Administration 47%, Medication Monitoring 45% and Role of the Pharmacist 44%. For independent variables that were associated with statistically significant at 0.05 (2-tailed) and Pearson correlation of moderate percentage included the High-Risk Medications 37% Medication Safety Strategies 36%, Standardization 34% and Education 32%. respectively. The independent variables were correlated with the dependent variable, except the Medication reconciliation which was the only one factor unrelated to administration error.

In addition to the afore-mentioned results, extra–study to find out the external impacts affecting the most common type of medication errors in a Thai hospital implying the certification standards of the hospital and the professional standard of hospital staffs was carried out. The two external impacts as independent variables and administration error as dependent variables were analysed by applying the Independent samples t-test. The certification standards of the hospital was shown in Table 6 and the professional standard of hospital staffs was shown in Table 7.

**Table 6:** The mean and standard deviation of the hospital certification standards in correlation with administration error

	The certification standards of the hospital	N	Mean	Std.Deviation	Std. Error	Mean	t-value	Sig.(2-tailed)
Administration errors	affect	42	4.50	0.506	0.078	7.476	<0.001
	not affect	4	2.50	0.577	0.289		

**Table 7:** The mean and standard deviation of the hospital staff professional standard in correlation with administration error

	The certification standards of the hospital	N	Mean	Std.Deviation	Std. Error	Mean	t-value	Sig.(2-tailed)
Administration errors	affect	5	2.40	1.517	0.678	-4.566	<0.001
	not affect	41	4.29	0.782	0.122		

By Levene's Test for Equality of Variances, most respondents recognized the certification standards of the hospital was affected by administration error.

By Levene's Test for Equality of Variances, most respondents realized the need for professional standard of hospital staff.

**Figure 4:**
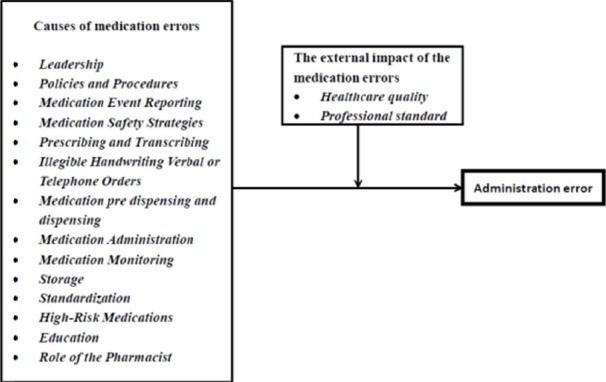
Framework of causes of administration error in Thai hospitals

The conclusion was that the most medication error type in the hospital studied was the administration error affecting on 14 key factors and 2 external impacts of the hospital except the medication reconciliation. According to this study, result in the hypothesis 3 can be summarized in the revised framework of the causes of administration error (Figure 4)

#### 4.3.4 Questions 4: What are the detail descriptions of these factors that affect the most common type of medication errors?

The detail descriptions particularly in Medication Administration factor from theory of ECRI Institute (2008) which contain 35 independent variables. The most common type of medication errors in the hospital from this study was the administration error which was the dependent variable. The entered/removed technique in regression analysis was applied to demonstrate the correlation of the above variables. Determination of the predictive relationship between the level of importance of the independent variables and the dependent variable was shown in Table 8.

**Table 8:** The result of multiple regression analysis

Model		Unstandardized Coefficients			Standardized Coefficients	Adjusted R Square	Sig.

		B	Std. Error	Beta
1	(Constant)	3.473	0.512		0.068	<0.001
	Patients’ identification (PI)	0.230	0.111	0.298		0.044
	(Constant)	4.232	0.578		0.161	<0.001
2	Patients’ identification (PI)	0.513	0.157	0.666		0.002
	Detect risk of adverse effects (AE)	0.456	0.188	0.495		0.020
	(Constant)	3.845	0.588		0.219	<0.001
	Patients’ identification (PI)	0.489	0.152	0.634		0.003
3	Detect risk of adverse effects (AE)	0.653	0.205	0.708		0.003
	Drug administration at the right time, dose and route (DA)	0.310	0.152	0.360		0.047

The multiple regression model 3 of this study produced R² = 0.271, F (3, 42) = 5.208, p < 0.05. As being shown in Table 8, the variables “Patients’ identification before administration of medication”, “detection of adverse medication effects” and “Assurance that the medication is administered at the right time, in the prescribed dose, and via the right route” had significant positive regression weights, Therefore, the multiple regression equation is as follows: Administration error = 3.845 + 0.489 PI+0.653AE+0.310DA.

**Figure 5:**
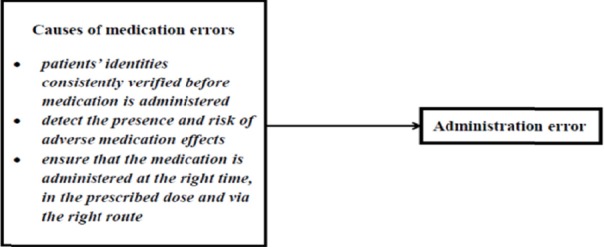
Framework of causes of administration error

The research question 4 can be summarized in the framework in Figure 5.

## 5. Discussion

Medication errors are preventable and can occur in any stage of the medication processes. Recognition of The causes can therefore prevent medication errors, resulting in patient safety. The results and findings of this study will be adapted to develop solutions preventing medication error in hospitals, especially the administration error which is most common in private hospitals in Thailand. Comparing the findings in Thai hospital with other countries, we found that all the important factors, except the medication reconciliation, have a linear relationship with administration error. As the medication reconciliation concept is still in preliminary stage of Thai hospital policy, it has not been applied in some hospitals. If this policy is utilized universally, medication reconciliation is expected to be in a linear relationship with administration error as in other countries. One of the failure to reduce medication errors in Thai hospital is the overwhelming effects of external impact such as the certification standard of the hospital and the professional standard of hospital staff. The medication errors should be decreased if these external impacts are properly managed.

This study also note that medication errors could be prevented in various measures, and the most common problem in medication processes is the administration errors in in-patient department. As human error is mainly involved in the administration errors, and the mode of human error modification is still not efficient, future research is needed to solve the problem. It requires design of a new system to reduce medication errors.

The author's personal concept is to adjust the human behavior, particularly with appropriate technology and effective management. The effective management is to plan for a responsible committee or body to set goals and procedures clearly and consistently to promote safety drug use for prevention of medication errors. The role, duties and responsibilities of each person should be definite. Working together to promote an interdisciplinary team with patient participation in conjunction with the utilization of various information technology systems would produce a beneficial outcome.
